# Experimental characterization and finite element investigation of SiO_2_ nanoparticles reinforced dental resin composite

**DOI:** 10.1038/s41598-024-58114-7

**Published:** 2024-04-02

**Authors:** Babak Jaleh, Mohammad Kashfi, Behnaz Feizi Mohazzab, Morteza Shakhsi Niaee, Fariborz Vafaee, Parisa Fakhri, Reza Golbedaghi, Rui Fausto

**Affiliations:** 1 Department of Physics, Faculty of Science, Bu-Ali Sina University, Hamedan, Iran; 2 Mechanical Engineering Department, Engineering Faculty, Ayatollah Boroujerdi University, Boroujerd, Iran; 3 Science and Technology Park, Qazvin, Iran; 4 Prosthodontics Dental Implants Research Center, School of Dentistry, Hamadan University of Medical Sciences, Hamedan, Iran; 5 Instrumentation Research Group, Niroo Research Institute (NRI), Tehran, Iran; 6 Department of Chemistry, Payame Noor University (PNU), Tehran, Iran; 7 Department of Chemistry, CQC-IMS, University of Coimbra, 3004-525 Coimbra, Portugal; 8 Department of Physics, Faculty of Sciences and Letters, Istanbul Kultur University, Ataköy Campus, Bakirköy, 34156 Istanbul Turkey; 9 Energy and Environment Research Group, Ayatollah Boroujerdi University, Boroujerd, Iran

**Keywords:** Nanocomposite, Dental resin, Finite element analysis, Mechanical properties, Mechanical engineering, Nanoparticles

## Abstract

In this study, a commercial dental resin was reinforced by SiO_2_ nanoparticles (NPs) with different concentrations to enhance its mechanical functionality. The material characterization and finite element analysis (FEA) have been performed to evaluate the mechanical properties. Wedge indentation and 3-point bending tests were conducted to assess the mechanical behavior of the prepared nanocomposites. The results revealed that the optimal content of NPs was achieved at 1% SiO_2_, resulting in a 35% increase in the indentation reaction force. Therefore, the sample containing 1% SiO_2_ NPs was considered for further tests. The morphology of selected sample was examined using field emission scanning electron microscopy (FE-SEM), revealing the homogeneous dispersion of SiO_2_ NPs with minimal agglomeration. X-ray diffraction (XRD) was employed to investigate the crystalline structure of the selected sample, indicating no change in the dental resin state upon adding SiO_2_ NPs. In the second part of the study, a novel approach called iterative FEA, supported by the experiment wedge indentation test, was used to determine the mechanical properties of the 1% SiO_2_-dental resin. Subsequently, the accurately determined material properties were assigned to a dental crown model to virtually investigate its behavior under oblique loading. The virtual test results demonstrated that most microcracks initiated from the top of the crown and extended through its thickness.

## Introduction

Teeth diseases pose a major health burden for many countries and affect people throughout their lifetime, causing pain, discomfort, and disfunction^1^. Over the years, scientists have been tackling this problem by introducing new composites that improve the efficiency of dental restorative materials, in particular to surpass the lack of mechanical stability^2^. It became evident that using mercury-containing composites or other inappropriate treatment materials may cause pernicious side effects on health, such as heart diseases and arthritis^3^. This fact stimulated the search for safer alternative materials^4,5^.

In the mid-1960s, dental resin composites (DRCs) were proposed as novel dental fillers in dentistry due to their compelling features, such as appropriate strength, low toxicity, adequate bonding with teeth, and biocompatibility^6–8^. Accordingly, conventional fillers such as amalgam alloys were replaced by resin composites, which are applied systematically worldwide in dental restorations^9^. DRCs typically consist of three major components: the resin-based matrix (organic matrix), the fillers (inorganic matrix), and a crosslinker (to enhance the chemical bonding between the filler and the organic matrix)^10^. The inorganic fillers are dispersed in the DRC to guarantee the appropriate physical and mechanical properties of the composite material, such as greater strength, wear resistance, decreased polymerization shrinkage, improved transparency, fluorescence and color, and a reduced exothermic reaction on polymerization^11^.

Different types of inorganic nanoparticles, such as SiO_2_, ZrO_2_, and Al_2_O_3_, have been proposed as dopants by the light curing method to improve the mechanical features of the DRCs^12^. Among them, the SiO_2_ particles have attracted great interest owing to low cost and unique characteristics, such as biocompatibility, stability in physical and chemical properties, facile surface functionalization, and adjustable particle size. Meanwhile, SiO_2_ particles can reduce polymerization volume shrinkage, resulting in the improvement of mechanical properties and the reduction of restorative fractures^13,14^.

In dental restoration, the high mechanical performances of dental materials are of great importance. The evaluation of mechanical behavior of DRCs under intraoral loading relies on the accurate measurement of the material mechanical properties. The finite element analysis (FEA) is widely used in the literature to virtually study the mechanical behavior of dental materials^15,16^ providing a broad set of possibilities for testing restorative materials that can be greatly extended to the clinical setting^17,18^. Nakano et al^19^ studied maximum fracture load of composite resin reinforced by silica-nylon. FEA was used for 3D modeling to identify the highest stress concentration that occurred. The effect of Silica-Nylon reinforcement on the fracture load and stress distribution of a dental prosthesis has been investigated by Firmini et al.^20^. The stress distribution for non-aged groups was simulated using FEA. Peskersoy et al^21^ evaluated and validated the nanomechanical properties of four composite and ceramic-based hybrid dental materials reinforced by silica and lithium disilicate to provide engineering tools for comparing the findings of the mechanical experimental data with the calculated theory of the finite element model. FEA was applied to investigate the stress distribution. Considering the special properties of dental resin materials filled with silica-based material, evaluation of mechanical properties of these materials is of special importance. Therefore, more investigations are needed to study the mechanical characteristics of silica reinforced DRCs.

**Figure 1:**
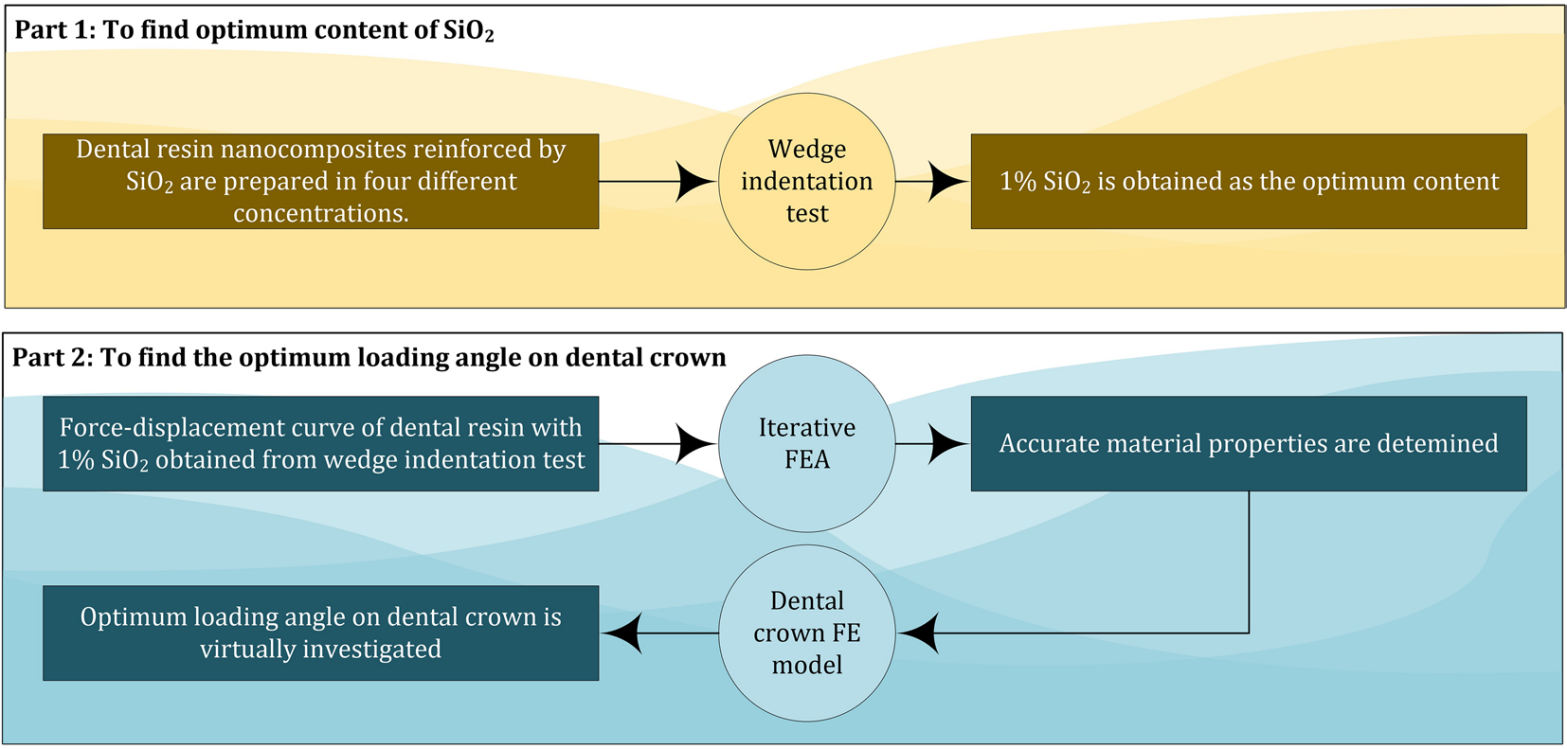
Research progress of this investigation.

The current study utilizes a new iterative simulation approach based on finite element (FE) modeling to assess the mechanical characteristics of a dental resin that has been reinforced with SiO_2_. This method eliminates the requirement for additional experimental testing, and the validated FE models are subsequently used to determine the optimal loading angle for a dental crown when subjected to oblique loading. Overally, the current investigation is divided into the following segments (refer to Figure 1):

Part 1: The wedge indentation test is utilized to determine the optimum content of SiO_2_ nanoparticles (NPs). The morphology and structure of the optimum fabricated nanocomposite are analyzed using SEM and XRD methods.
Part 2: The precise mechanical characteristics of the optimal sample obtained in Part 1 are calculated via an innovative technique called iterative FEA^22,23^. These properties are necessary for simulating a dental crown subjected to oblique loading. The optimal loading angle on the dental crown composed of the examined nanocomposite with the optimal concentration of SiO_2_ is evaluated virtually at various loading angles.

## Materials and experiments

### Synthesis of SiO_2_-reinforced DRC

In this study, the NextDent C&B resin from NextDent in Netherlands, which is intended for the manufacturing of 3D printed crowns and bridges, was utilized as the base material for preparing dental composites. The resin was reinforced with specified amount of SiO_2_ NPs (according to Table 1) through magnetic stirring to improve its mechanical stability, resulting in a nanocomposite labeled as “x%SiO_2_-dental resin”, where x is the concentration of SiO_2_ NPs. The sample with 0% SiO_2_ NP refers to the reference dental resin sample. The sample preparation was conducted according to the procedure suggested by the supplier, where 6 g of dental resin was stirred with SiO_2_ NPs (per required fraction) for 20 min in a dark environment to achieve a homogeneous mixture of resin and SiO_2_. The mixture was then poured into a plastic mold. The curing process involved the use of a handheld curing light (Guilin Woodpecker - LED.D Portable Curing Light, China) with a light intensity ranging from 650 to 800 mW/cm^2^. For the wedge test samples, curing was applied for 60 s on both sample sides, while for the 3-point bending specimens due to their size, it was extended to 120 s. These procedures were carried out in a controlled laboratory environment at room temperature.

**Table 1:** Amount of material used for the preparation of SiO_2_-dental resin nanocomposite.

SiO_2_%-resin%	SiO_2_ g-resin g
0.0–100.0	0.00–6.00
0.5–99.5	0.03–5.97
1.0–99.0	0.06–5.94
2.0–98.0	0.12–5.88

The crystalline structure was investigated through X-ray diffraction (XRD, Italstructure, ADP200, Italy and GNR explorer, Italy) in the 2θ range of 10–80 at the wavelength of 0.154 nm. High-resolution transmission electron microscopy (HRTEM, JEOL 2100, Tokyo, Japan) images were acquired to detect the morphology and structure of SiO_2_ NPs were performed to investigate nanocomposite constitutive elements. The morphology of reference dental resin and (b) 1% SiO_2_ -dental resin was investigated by FE-SEM (TESCAN-MIRAIII-SAMX, Czech Republic).

### Wedge indentation test

**Figure 2:**
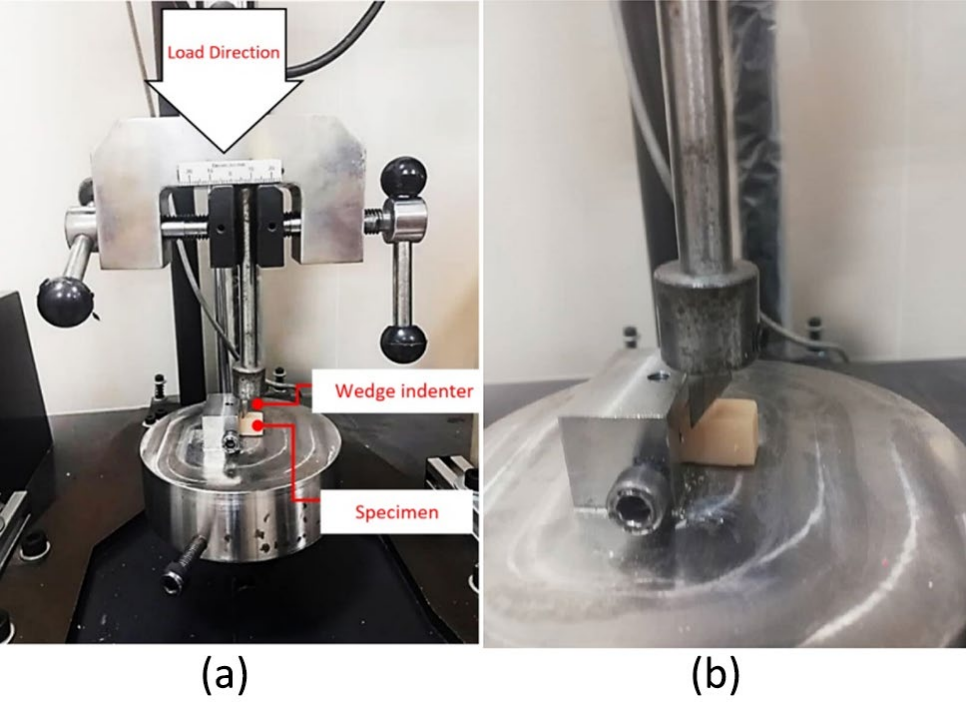
(**a**) The experimental setup of wedge indentation test, (**b**) the micro view of sample under the test.

The wedge indentation test evaluates material hardness and mechanical properties by applying a controlled force with a wedge-shaped indenter to create an indentation. It assesses the mechanical behavior of various materials and provides valuable insights into strength, toughness, and deformation resistance. The test involved clamping the sample in a fixture and applying a load using an indenter. This widely used test characterizes material properties and is especially useful for studying micro- or nanoscale mechanical behavior. SANTAM test machine with 50 kN capacity was employed with a loading rate of 0.5 mm/min to ensure quasi-static conditions. The sample dimensions were 10 × 10 × 25 mm. For each experimental group, three specimens were evaluated. The experimental setup is demonstrated in Fig. 2.

### 3-point bending test

**Figure 3:**
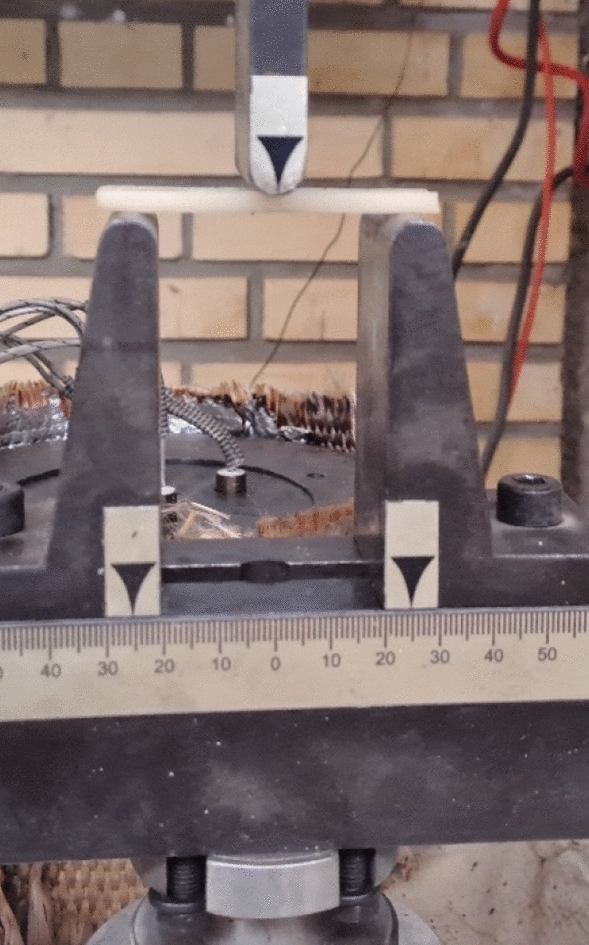
The experimental setup of 3-point bending test.

The 3-point bending test evaluates flexural properties such as strength and stiffness of materials by applying a load at the midpoint of a sample supported on two points. The 3-point bending test involves a beam supported at two points, with the load applied at the midpoint of the specimen. All tests were conducted following the guidelines specified by ASTM D790 and at room temperature and quasi-static condition with the rate of 0.5 mm/min using a SANTAM test machine. The prepared sample dimension was considered as 3.5 × 12.5 × 62.5 mm with a span of 50 mm according to the standard suggestion. Three specimens were tested to check the result repeatability. The test setup is shown in Fig. 3.

### Characterization of dental resins

The crystalline structures of the SiO_2_ NPs and the dental resin were assayed using an X-ray diffraction (XRD). High-resolution transmission electron microscopy (HRTEM, JEOL 2100) analysis was carried out to elucidate the morphology of SiO_2_ NPs. The morphology of the SiO_2_ nanocomposite was studied and visualized using field emission scanning electron microscopy (FE-SEM; TESCAN MIRA3-XMU).

## Finite element analysis

### Numerical wedge indentation

**Figure 4:**
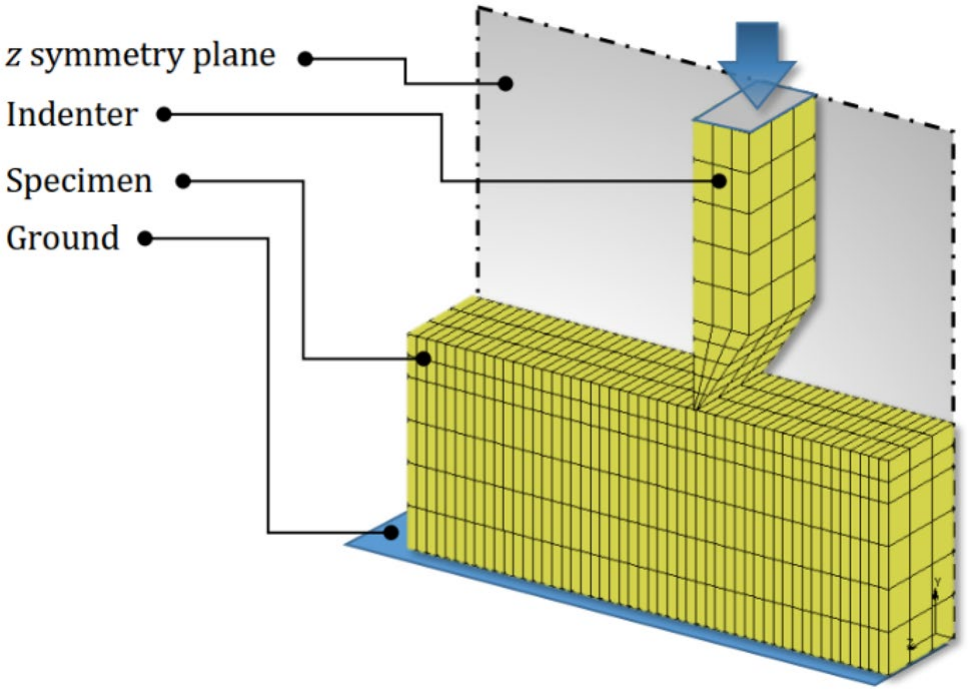
The FE model of wedge indentation test.

A numerical simulation of the wedge indentation test was performed using MSC Marc software as depicted in Fig. 4. The simulation model was composed of two distinct parts, namely the indenter and specimen. To reduce computational costs, a 1/2 model was created due to the model symmetry along the Z direction. A mesh convergence analysis was conducted, and the optimal number of elements and nodes for the entire model were determined to be 990 and 1506, respectively. An adaptive remeshing technique was employed during the simulation to improve model accuracy^24^. The remeshing algorithm was triggered under the indentation area, with a refinement criterion of 0.002 equivalent strain. The remeshing process was repeated twice to achieve convergence. The linear elastic material model was utilized for both the indenter and specimen based on their actual behavior. The mechanical properties of steel were assigned to the indenter model, with Young’s modulus of 200 GPa and Poisson’s ratio of 0.3^25^. The nodal displacements along the Y direction were fixed to define the ground plane, while displacements on the Z symmetry plane were fixed in the Z direction. In this study, instead of applying direct pressure, we utilized a contact interaction between two bodies (specimen and indenter) to apply a more realistic force. The contact interaction was defined between the surfaces of the two bodies in the simulation model.

### Virtual compression crown test under oblique loading

**Figure 5:**
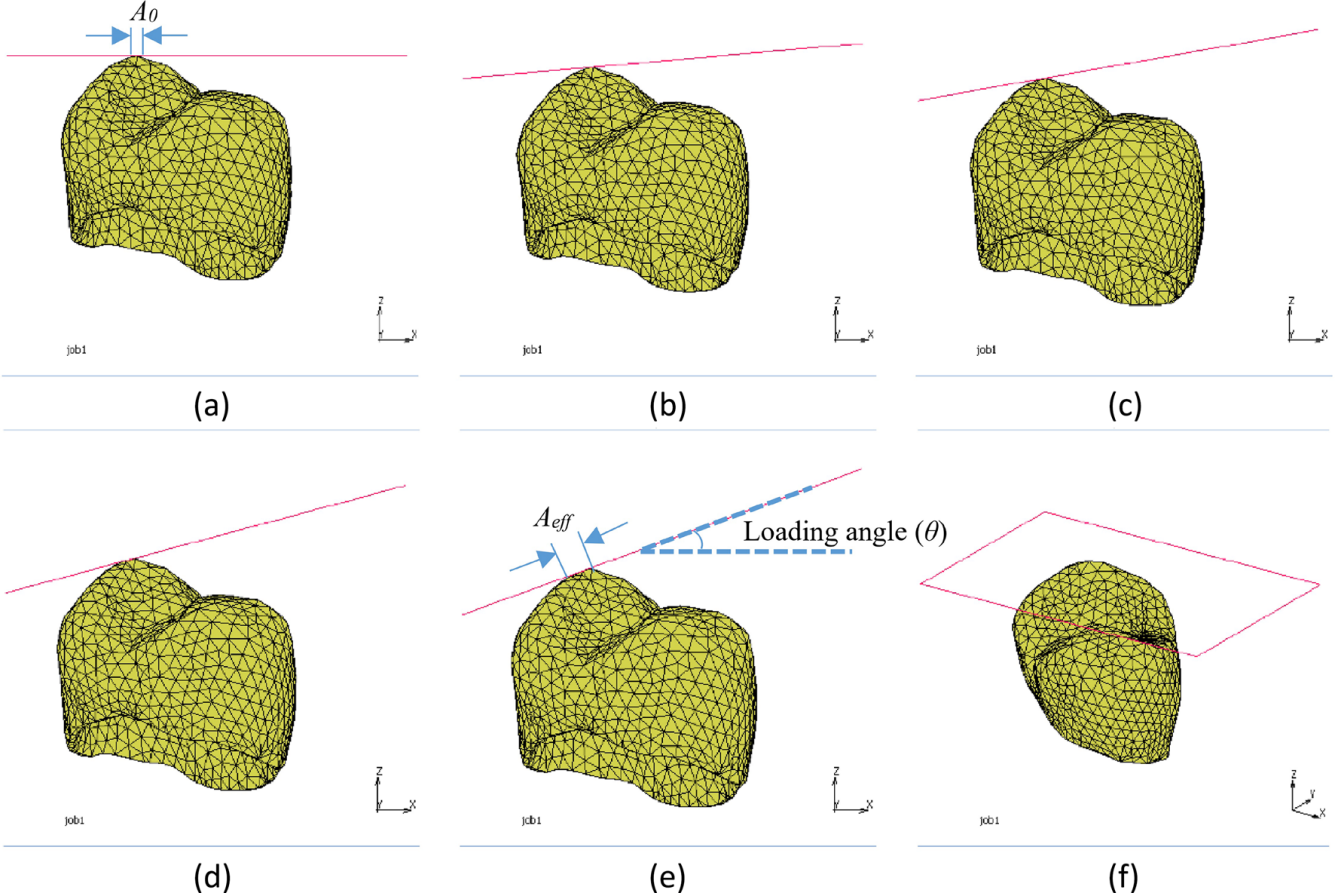
FE model of implant crow under oblique loading: (**a**) 0°, (**b**) 5°, (**c**) 10°, (**d**) 15°, (**e**) 20°, and (**f**) isometric view.

The study aimed to investigate the behavior of dental crowns under compressive loading through a virtual compressive crown test. The test involved applying a linearly increasing load to the crown’s top using a rigid-geometry element, while the inside of the crown was fixed by the implant abutment. The crown was designed and virtually tested specifically for an implant-supported restoration. To achieve convergence, an adaptive remeshing technique was employed, increasing the number of elements and nodes from 8384 and 2043 to 34755 and 42930. The simulations were performed on five different loading angles, ranging from 0 to 20°. The FE model construction is depicted in Fig. 5. The loading was applied using a rigid geometry plane, which was in contact with the crown’s top surface through a contact interaction. Additionally, to simulate the stable connection between the crown and the implant, nodal constraints were applied to the inner side of the crown, simulating the abutment contact. This constraint ensured that the inner side of the crown remained fixed during the loading simulation. It is worth noting the crown’s top point was considered as the rotation center, with the rotation axis defined along the Y direction. In this study, we used a moving plane to apply load at the cusp tip of the dental crown, evaluating its compressive behavior under different loading angles. This choice was based on multiple factors. Firstly, the cusp tip experiences common occlusal contact during chewing forces, making it a suitable location for load application. This allowed us to simulate a compressive load resembling typical occlusal contact. Secondly, although an oblique compressive masticatory load is more likely to occur at the internal transverse cusp ridge, this study aimed to assess the overall compressive behavior of the crown.

## Results and discussion

### Effect of SiO_2_ on the DRC mechanical properties

The results of wedge indentation test, depicted in Fig. 13, illustrate the relationship between the maximum reaction force and failure indention depth of the samples. The observed trend is consistent across all samples, with reaction force increasing linearly until the ultimate point, followed by an abrupt drop after material failure. This behavior is typical of brittle materials^26^.

**Figure 6:**
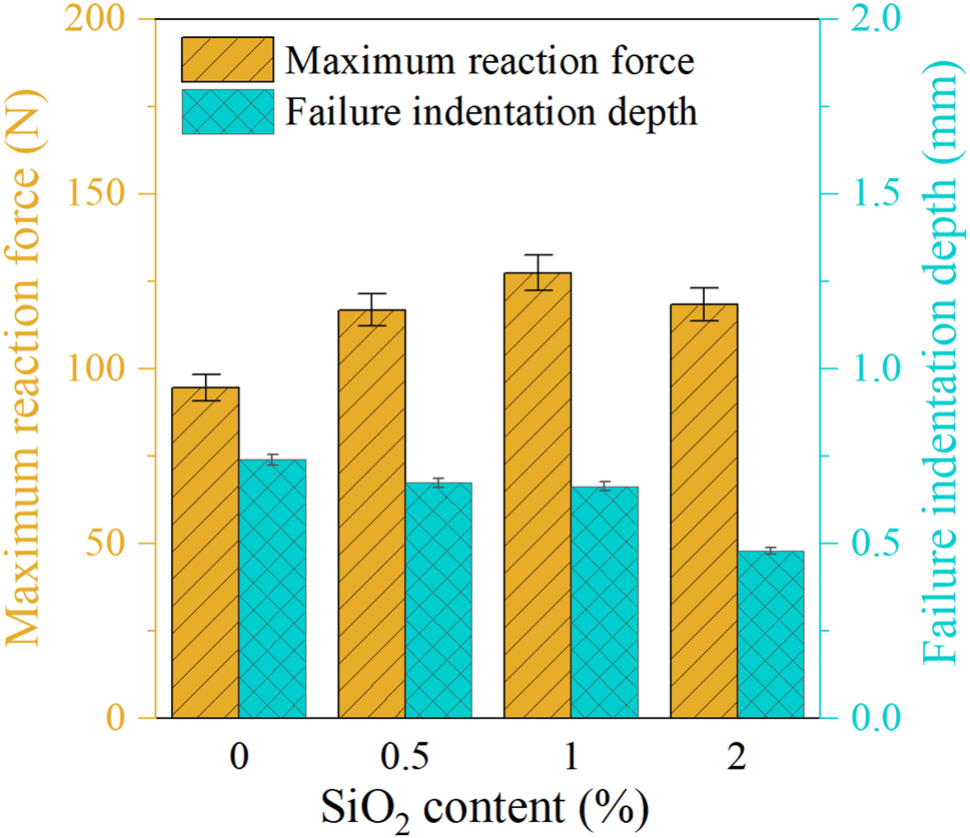
The effect of NPs content on the maximum reaction force and failure indentation depth obtained from the wedge indentation test.

As Fig. 6 shows the reference sample failed at an indention depth of 0.74 mm and a reaction force of 94.60 N. Increasing the SiO_2_ NPs content to 1% resulted in a significant increase of 35% in maximum reaction force; however, indention depth decreased by approximately 10% compared to the reference sample. Conversely, elevating SiO_2_ content up to 2% led to a decrease in both maximum reaction force and indention depth, indicating that this sample is more brittle and has less strength than the sample containing 1% SiO_2_ NPs, making it less suitable for intended applications. Nonetheless, the reaction force of the sample with 2% SiO_2_ is still 25% higher than that of the reference sample. Based on the experimental findings, it can be concluded that the optimal mass fraction of SiO_2_ NPs in the DRC is approximately 1%. Therefore, this sample was selected for further numerical investigation.

**Figure 7:**
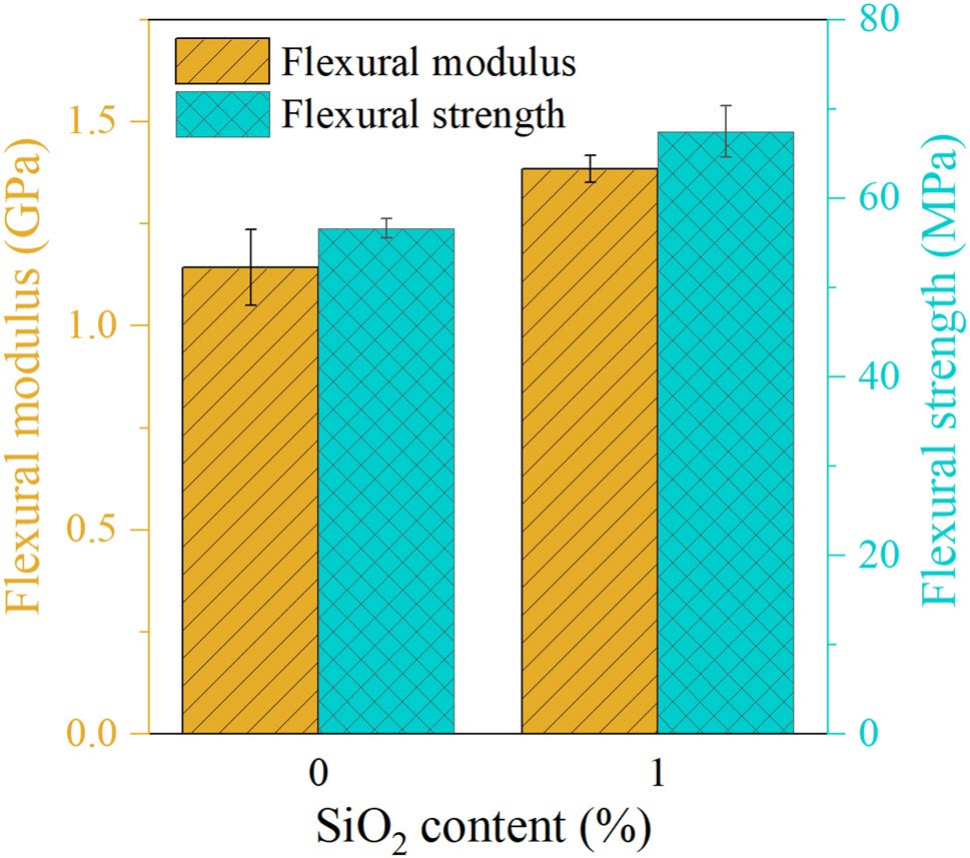
The effect of NPs content on the maximum reaction force and failure indentation depth obtained from the wedge indentation test.

Figure 7 demonstrates the effect of SiO_2_ content on the flexural modulus and strength of reference sample and 1%SiO_2_-dental resin samples. As the figure shows, increasing the SiO_2_ content up to 1% enhances the flexural modulus and strength by 21.15% and 19.20% respectively in comparison to the reference samples.

### Morphological characterization

**Figure 8:**
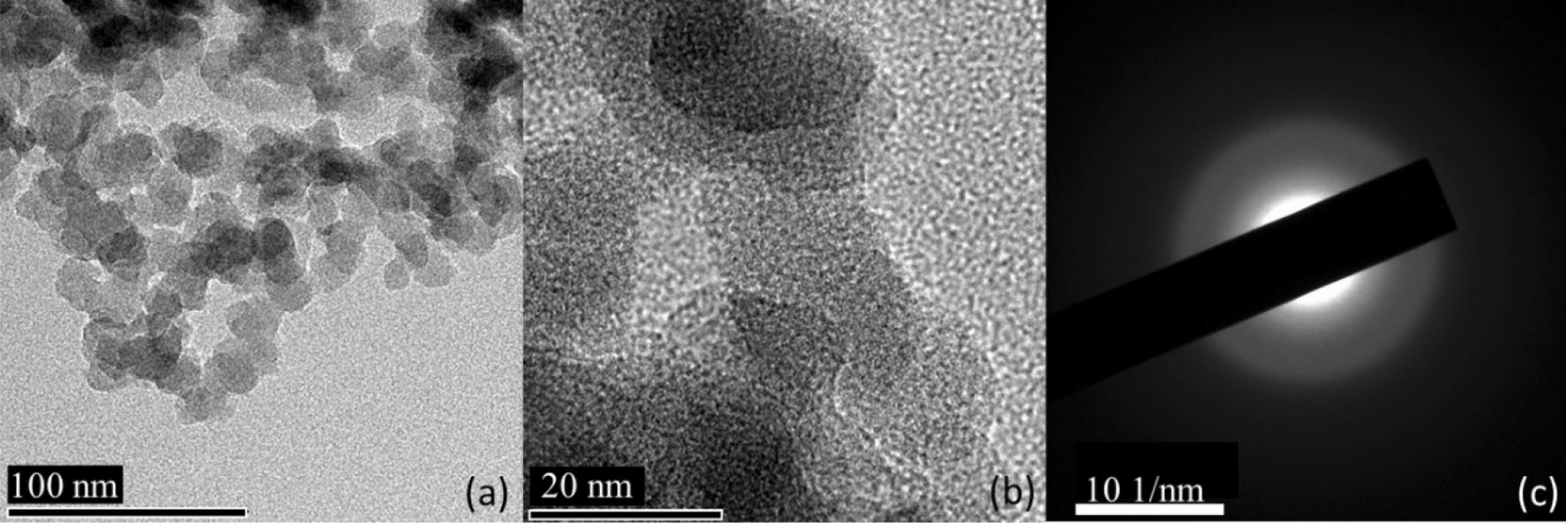
(**a**) and (**b**) HRTEM, and (**c**) SAED of SiO_2_ NPs.

The examination of SiO_2_ NPs using HRTEM and SAED techniques revealed important information about their structure. HRTEM images (Fig. 8a,b) showed that the SiO_2_ NPs had a spherical shape, which is a common morphology observed in nanoparticles. However, some areas showed aggregation, which can be due to the tendency of nanoparticles to stick together. The average diameter of the SiO_2_ nanoparticles was estimated to be 20 nm, which is a relatively small size for nanoparticles. This was determined by analyzing 50 NPs using ImageJ software, which is a widely used tool for particle size analysis. The SAED image in Fig. 8c showed diffused rings, which indicated the presence of amorphous material. Amorphous materials lack a long-range ordered structure and exhibit broad diffraction patterns in SAED. This suggests that the SiO_2_ nanoparticles had some degree of disorder in their structure.

Overall, the findings from the HRTEM and SAED analysis provide valuable insights into the structure of SiO_2_ nanoparticles. The spherical shape and small size of the nanoparticles make them attractive for various applications, while the presence of amorphous material highlights the need for further investigation into their properties and behavior.

### FE-SEM and XRD analysis

**Figure 9:**
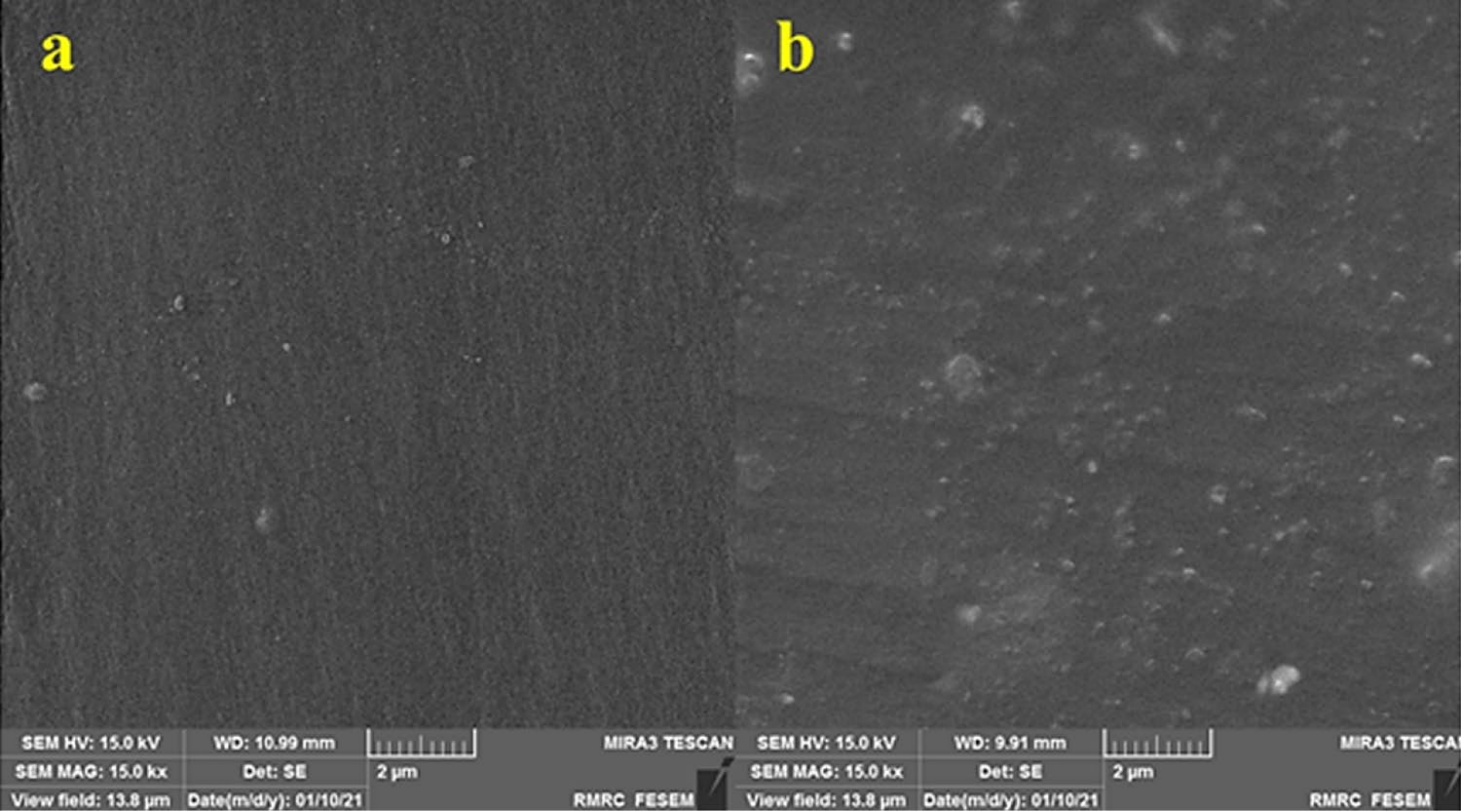
FE-SEM images of (**a**) reference dental resin and (**b**) 1% SiO_2_ -dental resin.

The morphologies of the reference dental resin and the nanocomposite containing 1% SiO_2_ were assessed through FE-SEM analysis. It is noteworthy that, based on the results of the wedge test, the nanocomposite with 1% SiO_2_ was found to be the optimal sample; therefore, the characterization outcomes of other samples were not reported. As shown in Figure 9, the surfaces of the 1% SiO_2_-dental resin are smooth and uniform, similarly to the reference sample, indicating that the SiO_2_ NPs were evenly dispersed throughout the matrix with no major agglomeration. However, some rough points on the surface were detected, which suggested minor agglomeration of the NPs during DRC synthesis. Notably, the introduction of SiO_2_ NPs did not cause any significant alterations in the material’s morphology. It is important to note that a smooth surface in DRCs is desirable in dentistry as it can help prevent bacteria growth. As such, the prepared SiO_2_-DRC appears to possess this advantageous property.

**Figure 10:**
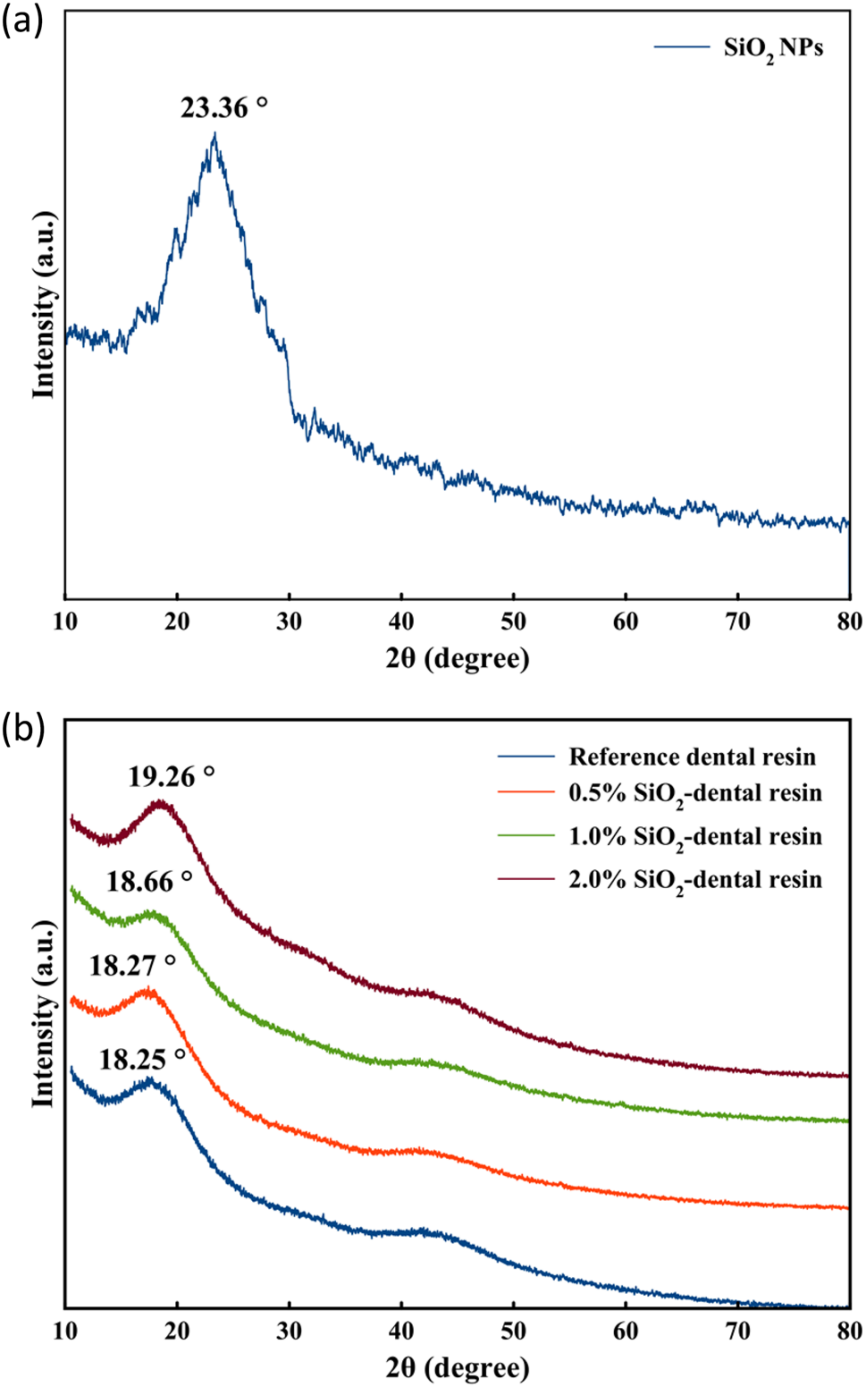
The XRD patterns of (**a**) SiO_2_ NPs, (**b**) SiO_2_-dental resin nanocomposites.

The crystalline structure of the SiO_2_ NPs, reference sample and 1% SiO_2_-dental resin were examined using XRD (refer to Fig. 10). As Fig. 10a shows the XRD pattern of SiO_2_ NPs displayed a broad feature typical of amorphous SiO_2_ at 2θ ≈ 23.36°^27,28^. Similarly, the dental resin reference sample exhibited broad features characteristic of an amorphous material without showing any narrow peak typical of a crystalline material (Fig. 10b). The incorporation of SiO_2_ NPs caused the resin peaks to shift towards higher angles, indicating that the SiO_2_ NPs were well integrated with the DRC. From comparing the XRD patterns of the reference sample and the 1%SiO_2_-dental resin, it can be inferred that the amorphous form of the prepared nanocomposite is structurally similar to the reference sample. Furthermore, no significant changes occurred in the state of the resin matrix upon introducing SiO_2_ NPs.

### Iterative FEA for calculating accurate material properties

The accuracy of FE models is heavily reliant on the input material properties utilized in the material model. To determine the precise values of the model mechanical properties, an iterative FE analysis based on optimization is required^29,30^. The wedge indentation test is selected as the most suitable test for this purpose since it reflects the behavior of the DRC.

In this method, the experimental setup is modeled in FE simulation software with identical boundary and test conditions. The experimental and numerical responses are then compared, and an objective function is developed to be minimized by an optimization algorithm. The present study compares the experimental reaction force-indentation depth curve obtained from the wedge indentation test with the numerically predicted curve. The root mean square (RMS) of error is considered the objective function, and the genetic algorithm (GA) is utilized as the optimization tool.

**Figure 11:**
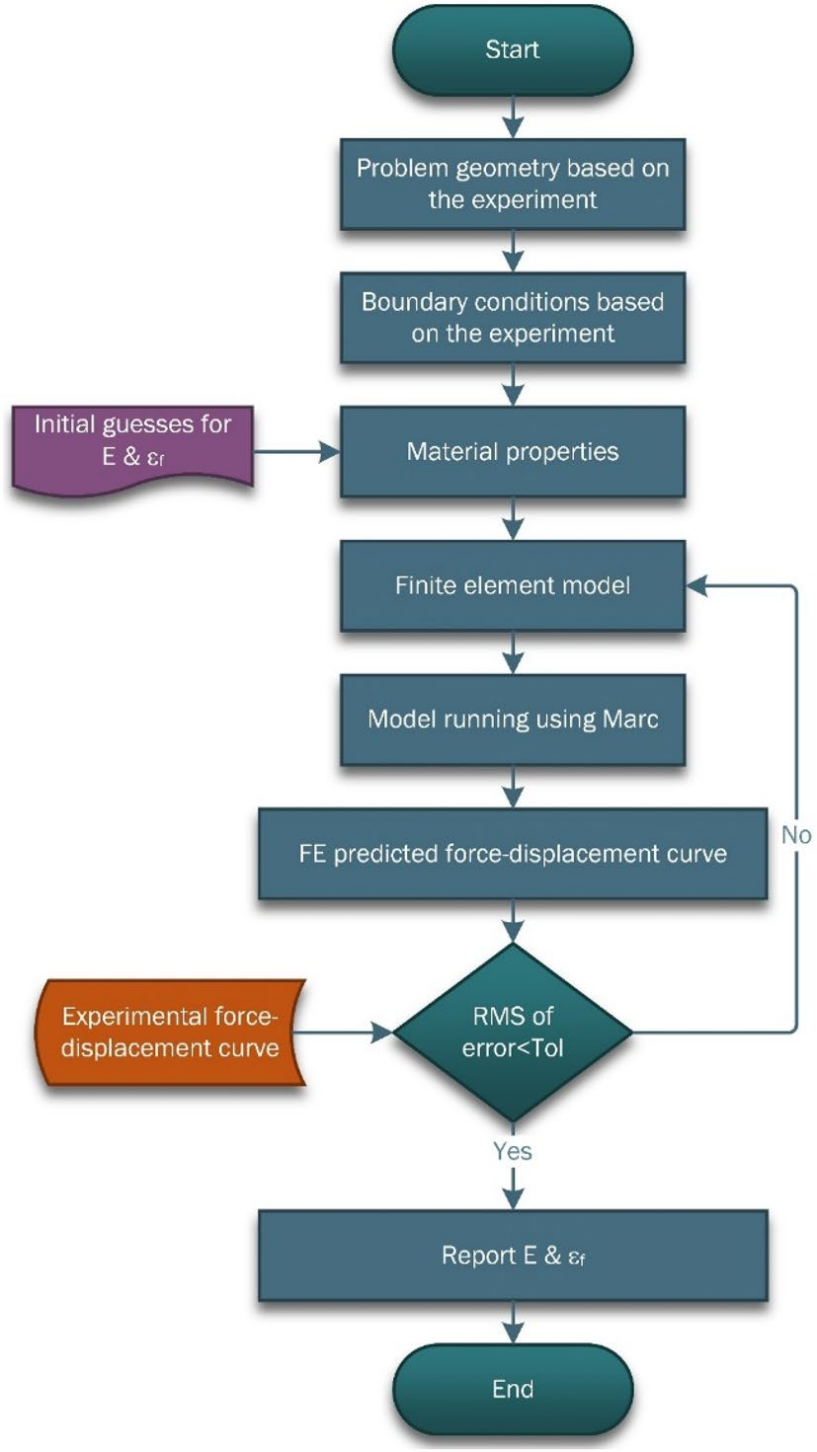
The optimization flow chart to determine the material properties using iterative FEA. **E** is the modulus of elasticity, **ε**_**f**_ is the material failure strain, **RMS** is the root mean square and **Tol** is the considered tolerance for converging the results.

The determination process is illustrated in Fig. 11. It begins by importing the geometry and boundary conditions according to the wedge indentation test. Young’s modulus and failure strain of the specimen are considered design variables of the optimization problem. At the start of the optimization process, an initial value of the design variables is considered based on the material datasheet, and the variation range of each parameter is altered to obtain a normal distribution in the input design variables. After the first simulation, the predicted curve is compared with the experimental curve, and the RMS error is calculated. Using the GA method, the design variables are iteratively modified to minimize the error based on a defined criterion. Once the calculated error meets the considered tolerance, the design variables are reported as the material mechanical properties. Young’s modulus and failure strain determined by the optimization process are 1.17 GPa and 6.02%, respectively. These material properties are assigned to the dental crown model to examine the stress analysis under oblique compressive loading.

### Stress analysis of crow under oblique loading

**Figure 12:**
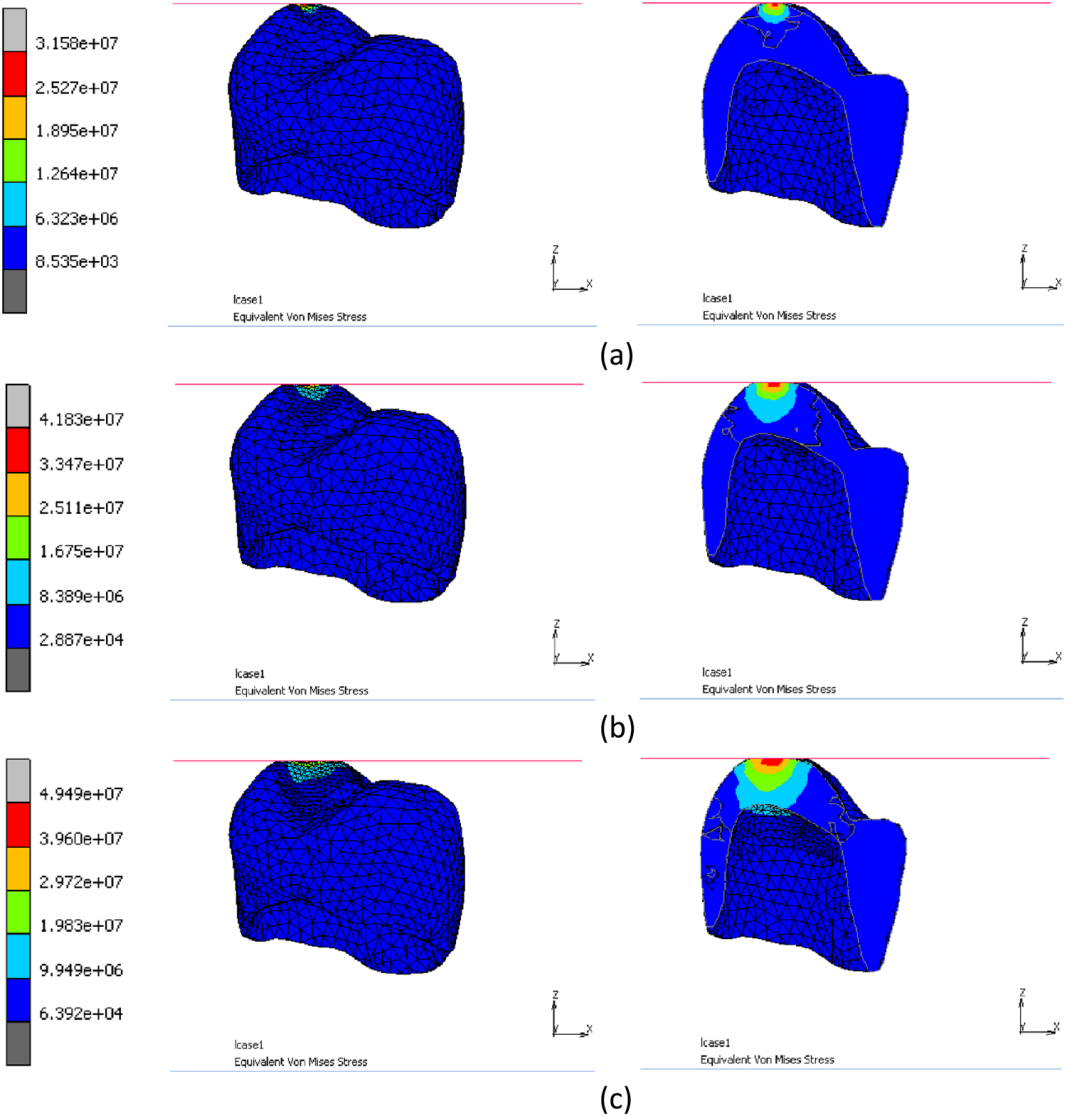
Von-Mises equivalent stress contour (full and split models) of the simulated crown at the simulation progress of (**a**) 20%, (**b**) 40% and (**c**) 60%.

To determine the compressive behavior of a dental crown containing 1% SiO_2_, the crown model was subjected to oblique loading through simulation. Figure 12 displays the von-Mises equivalent stress contour of the simulated crown, with both full and split models presented for better visualization. A cutting plane normal to the Y-axis was applied on the crown top point. The maximum value of equivalent stress increases with the increase in loading, as expected. The critical region of the crown for 0° compression loading is its top point, where microcracking is initiated and extends through the crown’s thickness.

### Effect of loading angle on the crown behavior

**Figure 13:**
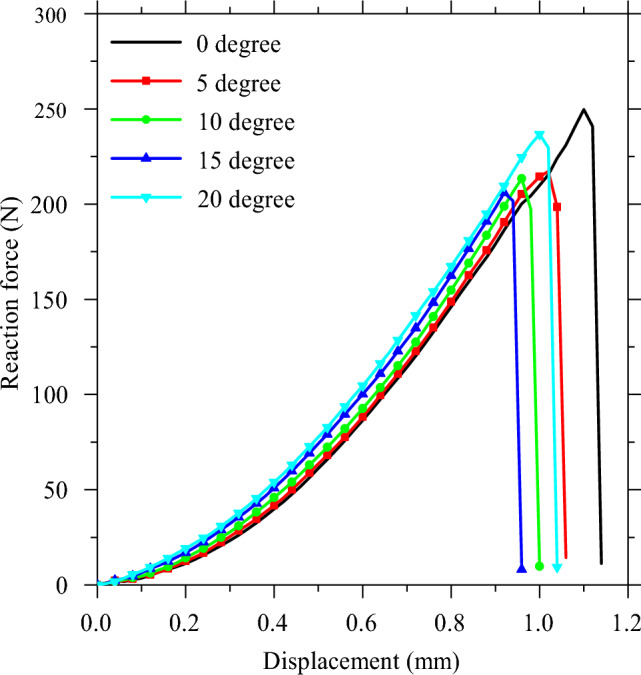
The comparison of the virtual force–displacement curve under different loading angles.

**Figure 14:**
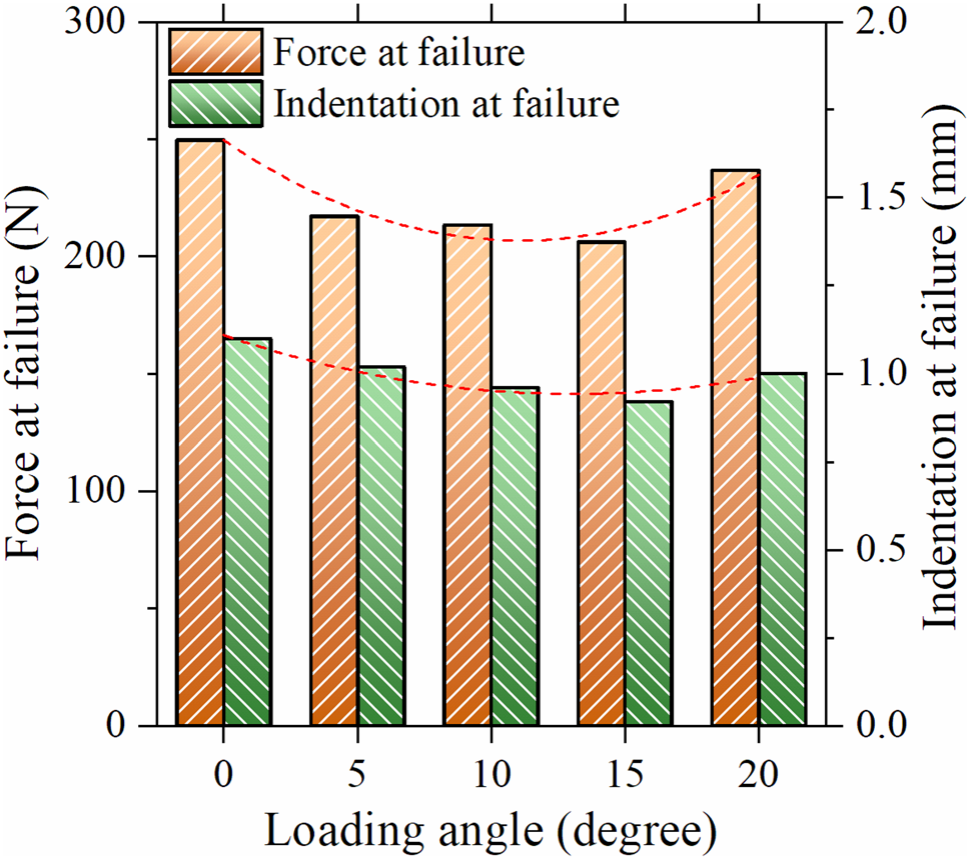
Failure force and indentation at failure for the simulated crown under different loading angles.

In order to determine the optimal loading angle for crown behavior, a virtual investigation was conducted. The loading angles of 0, 5, 10, 15 and 20° were simulated to evaluate the crown’s response under oblique loading. The reaction force versus displacement curve was recorded for each simulation, up to the point of structure failure. These curves are presented in Fig. 13. The results indicate that the maximum reaction force and failure displacement fell within the ranges of 206.05–249.62 N and 0.92–1.10 mm, respectively. The nonlinear nature of the crown geometry necessitated nonlinear calculations of material behavior. Figure 14 provides the reaction force and displacement at the point of structure failure.

The data depicted in the diagram indicates that an increase in the loading angle to 15° results in a decrease in the force at failure. Conversely, a higher loading angle causes an increase in the reaction force at failure. Specifically, the failure force at a loading angle of 20° was found to be 14.85% greater than that at 15°. Additionally, as illustrated in Fig. 5, the effective contact area (*A*_*eff*_) can be computed using the following formula:

1 $${A}_{eff}=\frac{{A}_{0}}{cos\left(\theta \right)}$$

where *A*_*0*_ is the effective contact area at the loading angle of 0° (Fig. 5a) and *θ* is the loading angle (Fig. 5e). To elaborate, when the loading angle is higher, the contact area becomes more extensive, resulting in lower stresses. Conversely, increasing the loading angle leads to higher shear force on the contact area and, consequently, greater shear stresses in that region. These opposing effects give rise to the concave trend seen in Fig. 14. By fitting a second-order polynomial curve to the data, a relationship was established between the failure force (F_f_) and the loading angle (θ) (Eq. 2). The same procedure was used to calculate the failure displacement (*D*_*f*_) (Eq. 3).

2 $$ F_{f} = 249.5 - 7.75\left( \theta \right) + 0.35\left( \theta \right)^{2} $$

3 $$ D_{f} = 1.11 - 0.025\left( \theta \right) + 0.00097\left( \theta \right)^{2} $$

The R^2^ values for Eqs. (2) and (3) were determined to be 0.9340 and 0.9379, respectively. Equation (3) indicates that the indentation at failure is mostly unaffected by the loading angle. Based on the analysis conducted, it can be inferred that when using the optimal SiO_2_-DRC, load-carrying capacity is diminished when the loading angle of the dental crown falls between 5 and 15°, as compared to a loading angle of 0°. Therefore, it is recommended that the dental abutment be modified to maintain a loading angle close to zero during typical usage. Clinical trials and longitudinal studies involving patients would provide more direct evidence and a better understanding of the clinical implications of maintaining a loading angle close to zero during typical usage. The clinical significance of our finding lies in the fact that during typical usage, dental crowns may be subjected to various loading angles when chewing or biting forces are applied. Our study suggests that if the loading angle deviates significantly from zero, the load-carrying capacity of the dental crown may be compromised.

## Conclusion

The initial phase of this study involved the preparation of dental composites using the light-curing method, with varying concentrations of SiO_2_ nanofillers, to enhance the mechanical properties. The wedge indentation test revealed that the optimal mass fraction of nanoparticles was found to be 1% SiO_2_ in which a significant increase of 35% in reaction force and a reduction of approximately 10% in indentation depth was observed compared to the reference sample. The morphology of the 1%SiO_2_-dental resin was investigated using FE-SEM, which demonstrated homogeneous dispersion of SiO_2_ NPs in the DRC with minimal agglomeration. The selected nanocomposites were also analyzed using XRD, revealing that there were no changes in the amorphous structures of SiO_2_ and resin matrix after mixing. In the second phase, a novel iterative FEA approach was utilized to determine the highly accurate material properties without the need for extra retardant experimental testing. By assigning these properties to a dental crown FE model loaded under various angles, a reasonable prediction of the dental crown’s behavior under oblique loading was obtained. The calculated results indicated that the dental crown performed best under 0° loading angle. The clinical significance of this finding is that a loading angle of 0° corresponds to a more favorable stress distribution and load-carrying capacity for the dental crown. This suggests that when the dental crown is subjected to forces during normal usage, such as chewing or biting, maintaining a loading angle close to zero would help optimize its performance and reduce the risk of failure.

## Data Availability

The data generated and analyzed during this study are available from the corresponding authors upon reasonable request.
